# Effects of Tall Fescue Endophyte Type and Dopamine Receptor D2 Genotype on Cow-Calf Performance during Late Gestation and Early Lactation

**DOI:** 10.3390/toxins13030195

**Published:** 2021-03-09

**Authors:** Sarah A. Wilbanks, Susan Maggie Justice, Thomas West, James L. Klotz, John G. Andrae, Susan K. Duckett

**Affiliations:** 1 Department of Animal and Veterinary Sciences, Clemson University, Clemson, SC 29634, USA; adams9@clemson.edu (S.A.W.); smj0059@auburn.edu (S.M.J.); Twest2@g.clemson.edu (T.W.); 2 USDA-ARS Forage Production Research Unit, Lexington, KY 40506, USA; James.klotz@usda.gov; 3 Department of Plant and Environmental Sciences, Clemson University, Clemson, SC 29634, USA; jandrae@clemson.edu

**Keywords:** fescue toxicosis, single nucleotide polymorphism, milk production, calf growth

## Abstract

Grazing endophyte-infected, toxic tall fescue reduces cow/calf production; therefore, this study examines alternate strategies such as use of novel endophyte fescue varieties during late gestation and early lactation or genetic selection of resistant cows. Pregnant cows (n = 75) were randomly assigned to fescue endophyte type: 1) endophyte-infected ergot alkaloid producing tall fescue (E+) or 2) novel endophyte-infected, non-toxic tall fescue (NOV) within maternal (A|A, n = 38 and G|G, n = 37) *DRD2* genotype to examine changes in cow/calf performance and milk production during late gestation and early lactation. Grazing E+ fescue pastures during late gestation reduced cow body weight gain but did not alter calf birth weight compared to NOV. Milk production and calf ADG during the first 30 day of lactation were lower for E+ than NOV. The calving rate was reduced, but not calving interval for E+ cows. The adjusted 205-day weight of calves was lower in those grazing E+ with their dams compared to NOV. There were no interactions between *DRD2* genotype and fescue endophyte type indicating that genotype was not associated with response to E+ fescue in this study. Overall, grazing NOV tall fescue pastures rather than E+ during critical stages of production improved cow gain during late gestation, calving rate, early milk production and calf growth.

## 1. Introduction

In the southeastern United States, tall fescue [*Lolium arundinaceum* (Schreb.) Darbysh; *Schedonorus phoenix* (Scop.) Holub] is the dominant cool season, perennial forage available for cow/calf production systems [1,2,3]. Kallenbach [2] estimates that about 12 million beef cows graze tall fescue in this region with reproductivea and liveweight losses estimated at USD 267 per cow in 2015. Most tall fescue contains an endophyte (*Epichloë coenophialia*) that produces ergot alkaloids, a class of mycotoxins that aid the plant in establishment and drought tolerance but is the causative agent for fescue toxicosis when ingested in livestock [1,2,3,4,5,6,7]. Fescue toxicosis is a syndrome that includes vasoconstriction, high body temperature, low heart rate, increased blood pressure, low serum prolactin, agalactia, reduced forage intake, and poor weight gains observed in livestock grazing endophyte-infected, ergot alkaloid producing tall fescue [3,5,8]. Ergovaline is mostly responsible for vasoconstriction of vasculature in beef cattle consuming endophyte-infected tall fescue [8]. Grazing endophyte-infected tall fescue reduces cow reproductive rates [9,10,11], lactation and calf weaning weights [3,5,12,13]. Varieties of tall fescue containing novel endophytes are now available that do not produce ergot alkaloids [14] but have a similar agronomic response as E+ [15,16] and are able to persist under grazing conditions [17]. Research has shown that grazing novel tall fescue instead of toxic endophyte-infected (E+) improves calf birth [18] and weaning weights [18,19], and stocker cattle performance [20,21]. Roberts and Andrae [3] suggested that one solution is to renovate and replace toxic tall fescue pastures with novel endophyte varieties; however, producer adoption of the novel varieties has been slow. Others have shown that grazing novel varieties during critical stages of production can improve overall performance and profitability in beef cow/calf production systems [19]. Critical stages in cow/calf production are during the last trimester of gestation when over 75% of fetal growth occurs [22], early lactation when calf growth rates are the highest [23,24], and rebreeding to avoid the negative impact of fescue toxicosis on reproduction [9,10,11].

**Figure 1:**
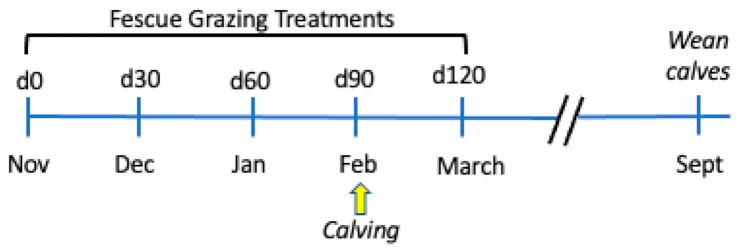
Timeline for the experimental study. Pregnant cows grazed NOV or E+ pastures during last trimester of gestation (90 days) and early lactation (30 days after calving).

Some have suggested there may be a genetic association in the cow population that allows producers to select for cows that are tolerant to the effects of fescue toxicosis [5,25,26]. For animals ingesting ergot alkaloids, a classic response is decreased serum prolactin concentrations [10,11,12,13,18,19] and vasoconstriction [3,5,8]. Ergot alkaloids are dopamine agonists that interact with *DRD2* in a competitive manner [27] to alter serum prolactin [28] and vasoconstriction [5,6,7,8]. Campbell et al. [29] identified a SNP in dopamine receptor D2 (DRD2) and established a relationship with serum prolactin concentrations in grazing steers and cows. They identified the G|G genotype in steers as having a greater reduction in serum prolactin values than A|A when grazing E+ fescue pastures. They suggested that the A|A genotype may have resistance to fescue toxicosis. Therefore, we chose to evaluate the *DRD2* SNP in cattle to examine if there is any genetic association with response to fescue toxicosis. The objectives of this study were to evaluate: (1) the use of novel, non-ergot alkaloid producing tall fescue (NOV) compared to E+ fescue pastures during the last trimester of gestation (90 days) and early lactation (first 30 days; Figure 1) on cow/calf performance and milk production, and (2) if maternal genotype for the *DRD2* SNP interacted with the response to grazing tall fescue by endophyte type.

## 2. Results

**Figure 2:**
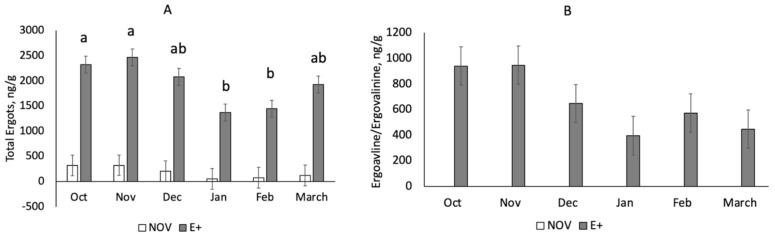
Total ergot alkaloid concentrations (**A**) or ergovaline/ergovalinine (**B**) for novel (NOV) or toxic (E+) endophyte-infected tall fescue pastures by month during time period when endophyte treatments were applied (year 1). Total ergot alkaloids differ (*p* = 0.049) by month but the interaction with fescue endophyte type was non-significant (*p* = 0.40). ^a,b^ Means with uncommon superscripts differ (*p* < 0.05). Ergovaline and ergovalinine concentrations did not differ (*p* = 0.47) by month or the interaction (*p* = 0.27) between endophyte type and month.

Total ergot alkaloid concentration (year 1) differed by fescue type (*p* < 0.0001) and month (*p* = 0.022) but the interaction was non-significant (*p* = 0.40). Pastures with E+ fescue had higher (*p* < 0.001) total ergot alkaloid levels compared to NOV during the endophyte type treatment period (Table 1). Total ergot alkaloid concentrations were higher (*p* < 0.05) in October and November than January and February (Figure 2A). Hay samples, which were fed in times when forage was limited, also had higher (*p* < 0.001) total ergot alkaloid levels for E+ than NOV (Table 1). In year 2, pasture and hay samples were examined using LC-MS/MS to quantify ergovaline and ergovalinine, which are mostly responsible for vasoconstriction [5,6]. Ergovaline and ergovalinine concentrations differed by endophyte type (*p* < 0.0001) but were non-significant by month (*p* = 0.83) or the interaction between endophyte type and month (*p* = 0.16; Figure 2B). Ergovaline and ergovalinine concentrations were greater (*p* < 0.001) in E+ pastures than NOV during the months when the endophyte type grazing experiment was conducted. Hay samples from E+ bales contained greater (*p* < 0.001) ergovaline and ergovalinine concentrations compared to NOV.

**Figure 3:**
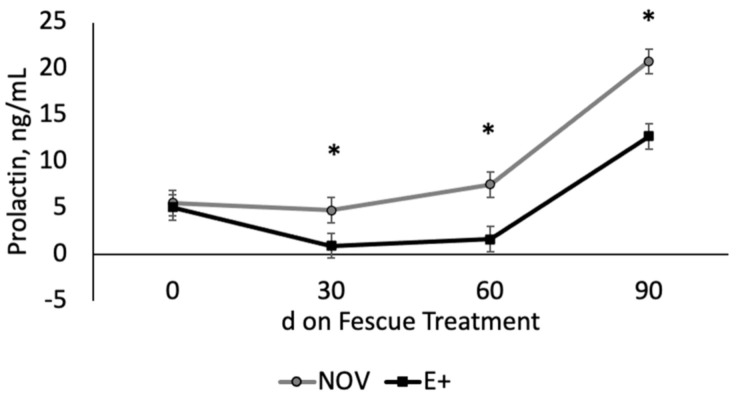
Serum prolactin concentrations in cows grazing endophyte-infected ergot alkaloid producing (E+) or novel endophyte-infected (NOV) during the last trimester of gestation by time on treatment. The interaction between endophyte type and time on fescue treatment was significant (*p* = 0.0088). * Denotes differences between endophyte treatments at that time point (*p* < 0.05).

**Figure 4:**
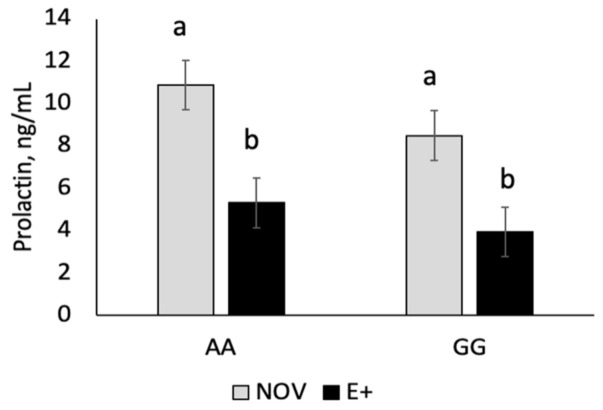
Maternal *DRD2* genotype for serum prolactin concentrations in cows grazing endophyte-infected ergot alkaloid producing (E+) or novel endophyte-infected (NOV) averaged over the last trimester of gestation. The interaction between endophyte type and maternal genotype, maternal genotype and time on fescue, or endophyte type, time on fescue, and maternal genotype were all non-significant (*p* > 0.05). ^ab^Means with uncommon superscripts differ (*p* < 0.05).

The Angus cow herd (n = 227; 3–7 years of age) at the Piedmont Research and Education Center was genotyped for *DRD2* SNP [27]. The cow herd had a distribution of 24% A|A, 56% A|G, and 20% G|G. For this study, we used the homozygote *DRD2* genotypes of A|A (resistant) and G|G (susceptible) to assess if there is any related genetic resistance to fescue toxicosis during late gestation and early lactation. Serum prolactin concentrations were measured during time on endophyte type grazing treatment prior to calving. The two-way interaction between endophyte type and time on fescue was significant (*p* = 0.0088; Figure 3). All other two-way and three-way interactions were non-significant (*p* > 0.38). Serum prolactin concentrations were similar at the start of the grazing study (day 0). Serum prolactin concentrations were lower (*p* < 0.05) for E+ compared NOV after 30, 60 and 90 days on fescue treatment. Serum prolactin concentrations increased (*p* < 0.05) during the last 30 days of the grazing study in both NOV and E+; however, concentrations were lower for E+ than NOV. Serum prolactin concentrations were lower (*p* = 0.047) for cows with G|G versus A|A *DRD2* genotypes regardless of endophyte type during the grazing study (Figure 4).

**Figure 5:**
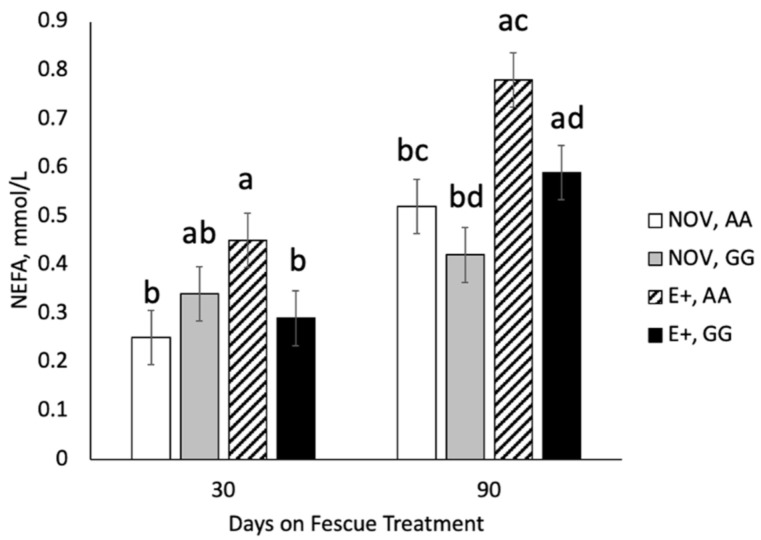
Serum non-esterified fatty acids (NEFA) in cows grazing endophyte-infected ergot alkaloid producing (E+) or endophyte-infected non-ergot alkaloid producing (NOV) during the last trimester of gestation. The interaction between maternal *DRD2* genotype and fescue endophyte type was significant (*p* = 0.017) on day 30 of fescue endophyte treatment. Endophyte type (*p* = 0.0002) and maternal *DRD2* genotype (*p* = 0.010) were significant but the interaction was non-significant on day 90 of fescue endophyte treatment. ^a,b^ Means with uncommon superscripts differ (*p* < 0.05) by endophyte type. ^c,d^ Means with uncommon superscripts differ (*p* < 0.05) by maternal genotype.

**Figure 6:**
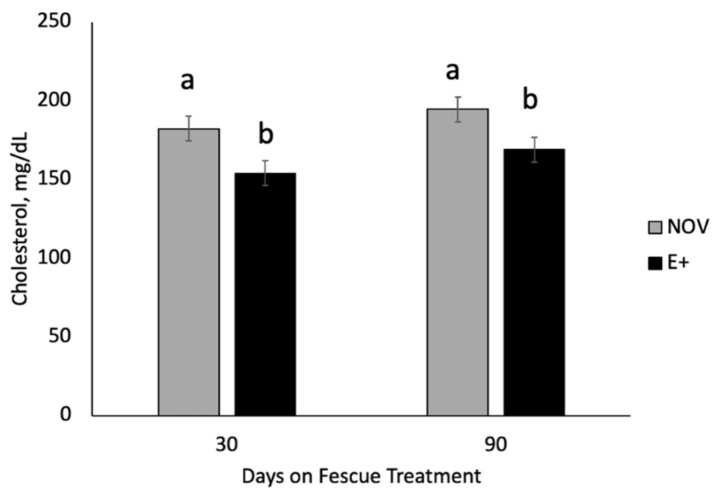
Serum cholesterol concentrations in cows grazing endophyte-infected ergot alkaloid producing (E+) or endophyte-infected non-ergot alkaloid producing (NOV) at 30 and 90 days on fescue endophyte treatments during the last trimester of gestation. All interactions were non-significant (*p* > 0.05) and main effects are shown in the figure for each time. ^a,b^ Means with uncommon superscripts differ (*p* < 0.05) at each time of sampling.

Serum non-esterified fatty acids (NEFA) and cholesterol concentrations were also examined during the fescue endophyte grazing treatment. At d 30 of endophyte treatment, the interaction between maternal genotype and fescue endophyte treatment was significant (*p* = 0.017) (Figure 5). Cows with A|A genotype had elevated serum NEFA concentrations when grazing E+ tall fescue compared to NOV. Cows with G|G genotype had similar serum NEFA concentrations when grazing the different endophyte types. At 90 d on treatment, serum NEFA concentrations were higher (*p* = 0.0002) in cows grazing E+ compared NOV regardless of genotype. Serum NEFA concentrations at d 90 were greater (*p* = 0.01) for A|A than G|G genotypes regardless of fescue endophyte treatment. The interaction between genotype and fescue treatment was non-significant (*p* = 0.47) for serum NEFA on d 90. Serum cholesterol concentrations differed (*p* < 0.05) by fescue endophyte type but did not differ by maternal *DRD2* genotype (*p* > 0.37) or interaction (*p* > 0.59) on day 30 and d 90 of fescue endophyte treatment. Serum cholesterol concentrations were greater (*p* = 0.0027) in cows grazing NOV compared to E+ fescue pastures (Figure 6) on day 30 and 90 of fescue endophyte treatment.

**Figure 7:**
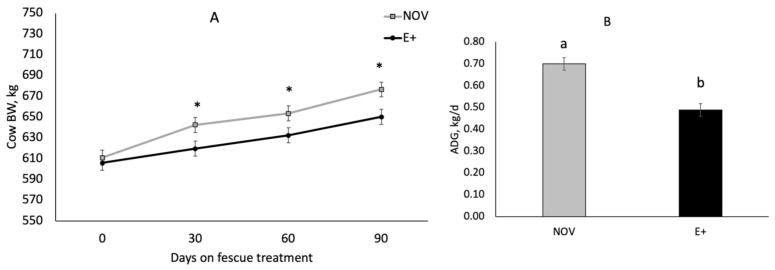
Changes in cow body weight (BW; **A**) and average daily gain (ADG; **B**) of cows grazing endophyte-infected ergot alkaloid producing (E+) or endophyte-infected non-ergot alkaloid producing (NOV) during last gestation. Interactions between endophyte type, year, and maternal DRD2 genotype were non-significant for body weight gain and average daily gain. * Denotes differences (*p* < 0.05) in body weight by endophyte treatment. ^a,b^ Means with uncommon superscripts differ (*p* < 0.05).

Cow body weight (BW) was measured over time during the fescue endophyte grazing study. Cow body weight differed by endophyte treatment (*p* = 0.0019) and time on fescue (*p* < 0.001) but the interaction was non-significant (*p* = 0.45). Cow body weight increased over time (*p* = 0.001) during late gestation. Cow BW was lower (*p* = 0.0019) in cows grazing E+ fescue compared to NOV after 30, 60, and 90 of grazing (Figure 7A). There was a trend (*p* = 0.054) for G|G cows to be heavier than A|A cows but there were no significant interactions with genotype and other variables (*p* > 0.34). Average daily gain during the last trimester of gestation differed by endophyte type (*p* = 0.0012) but maternal genotype and all interactions were non-significant (*p* > 0.30). Cows grazing E+ fescue pastures had 30% lower (*p* = 0.0012) average daily gains during the last trimester of gestation compared to NOV (Figure 7B).

**Figure 8:**
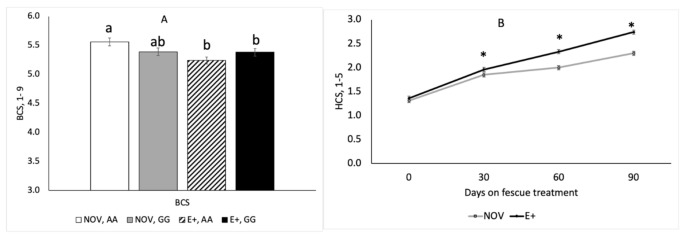
Body condition score (BCS; **A**) and hair coat scores (HCS; **B**) by fescue endophyte type during the last 90 days of gestation. The interaction between endophyte type and maternal genotype was significant for BCS. ^a,b^ Means with uncommon superscripts differ (*p* < 0.05). The interaction between endophyte type and time on fescue was significant for HCS. * Denotes significance (*p* < 0.05).

The interaction between endophyte type and maternal *DRD2* genotype was significant (*p* = 0.012) for cow body condition score (BCS). Cows with A|A genotype had lower (*p* < 0.05) BCS when grazing E+ fescue than NOV; however, for G|G cows there was no difference in BCS by fescue endophyte type (Figure 8A). Ultrasound measurements of rump fat thickness and rump muscle depth were obtained at the start and end of the fescue grazing experiment. The change in rump fat thickness did not differ by genotype (*p* = 0.41) or fescue endophyte type (*p* = 0.71) but the change in rump muscle depth was greater (*p* = 0.036) for cows grazing E+ fescue than NOV, regardless of genotype. The interaction between endophyte type and time on fescue was significant for hair coat score (HCS). Hair coat scores did not differ by maternal genotype (*p* = 0.35) or all interactions with maternal genotype were non-significant (*p* > 0.48). Hair coat scores increased (*p* = 0.041) over time in cows grazing E+ fescue and values were greater compared to NOV at day 60 and 90 of treatment (Figure 8B).

Interactions between year, endophyte type and maternal genotype were non-significant (*p* > 0.37) and the main effects are shown in Table 2. Dystocia scores did not differ (*p* > 0.05) by year, fescue endophyte treatment or maternal genotype. Calving interval from year 1 to year 2 did not differ (*p* > 0.05) by fescue endophyte type or maternal genotype. The calving rate was lower (*p* = 0.012) in cows grazing E+ than NOV. Calf birth weight nor adjusted calf birth weight did not differ (*p* = 0.63) by fescue endophyte treatment. Calf birth weight and adjusted calf birth weights were higher (*p* = 0.018) for A|A than G|G cows and by year with heavier (*p* = 0.055) calves in year 1. Calf sex did not differ (*p* > 0.05) by year, fescue endophyte type or maternal genotype. Cow milk production was estimated using the weigh-suckle-weigh procedure at 30 days after calving. Milk production was 15% lower (*p* = 0.047) in cows grazing E+ fescue compared to NOV. Milk production was higher (*p* = 0.014) in G|G than A|A cows. The interaction between fescue treatment and genotype was non-significant (*p* = 0.99).

**Figure 9:**
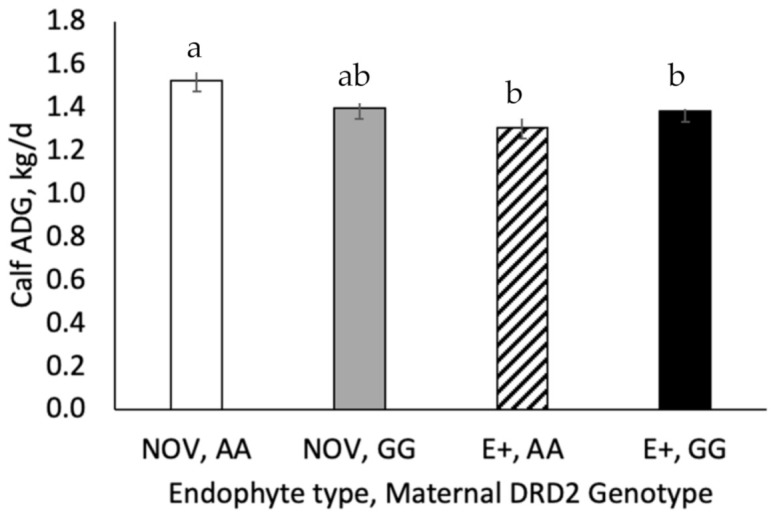
Calf average daily gain during the first 30 days of lactation in cows grazing different fescue endophyte type by maternal DRD2 genotype. The interaction between endophyte type and maternal DRD2 genotype was significant (*p* = 0.036) and simple effects are shown in the figure. ^a,b^ Means with uncommon superscripts differ (*p* < 0.05).

The interaction between fescue endophyte type and maternal genotype was significant (*p* = 0.036) for calf average daily gain during the first 30 days of lactation. Calf average daily gain (ADG) during the first 30 days of lactation was higher (*p* = 0.036) for calves born to A|A cows grazing NOV than G|G cows grazing NOV or E+ fescue (Figure 9). Calf weaning weight and adjusted calf weaning weights did not differ by fescue endophyte type (*p* > 0.52), genotype (*p* > 0.24) or the interaction (*p* > 0.94). Calf weaning weight and adjusted weaning weights were higher (*p* = 0.021) in year 1 compared to year 2. When weaning weight was calculated on an age adjusted basis, the adjusted 205 days weaning weights tended (*p* = 0.073) to be higher by 8.4 kg for NOV compared E+. Maternal genotype did not alter (*p* > 0.47) adjusted 205 days weaning weight and the interaction with fescue endophyte type was non-significant (*p* = 0.95 & 0.86). Adjusted 205 days ADG of the calves tended to be greater (*p* = 0.072) for NOV than E+.

## 3. Discussion

In the current study, stockpiled tall fescue with either endophyte-infected ergot alkaloid producing (E+) or novel endophyte-infected nontoxic (NOV) pastures were utilized for grazing during the last trimester of gestation (90 days) and early lactation (30 days) in a spring-calving Angus cow herd. In year 1, total ergot alkaloid was examined during the time period when fescue endophyte type treatments were applied (October–March). Total ergot alkaloid concentrations decreased from November and December to January and February. In year 2, a LC/MS analysis was developed to determine specifically ergovaline and ergovalinine levels in forage samples because they have been shown to be the causative agents of fescue toxicosis [5,8]. The concentrations of ergovaline reported in this study are at threshold levels (300–500 ppb) where fescue toxicosis can occur [31]. Kallenbach et al. [32] reported reduced ergovaline concentrations of stockpiled tall fescue from December to March. Curtis and Kallenbach [33] showed that ergovaline concentrations declined 50% in an 84-day period with stockpiling of tall fescue during the winter months. Ergot alkaloid concentration in E+ hay was lower than in the E+ pastures. Similarly, Roberts et al. [34] found ergovaline concentrations in hay are quickly reduced within the first 3 day after mowing and decline gradually during additional months of storage. The levels of ergovaline/ergovalinine in the current study are similar to those reported by Peters et al. [12] and Kallenbach et al. [32] but the total ergot alkaloid values are higher than those published by others for cow grazing E+ fescue [18,19,33].

Pregnant cows grazed E+ or NOV fescue pastures during critical stages of production, last trimester of gestation and the first 30 days of lactation. The interaction between fescue endophyte type and maternal *DRD2* genotype was non-significant and main effects of fescue endophyte type over time and maternal *DRD2* genotype are shown in Figure 3 and Figure 4. Before grazing treatments started (day 0, start of the last trimester of gestation), serum prolactin concentrations were similar. After 30, 60, and 90 days on fescue pastures, serum prolactin concentrations were lower for E+ than NOV. Serum prolactin levels, examined at day 90 of treatment, increased in both E+ and NOV compared to pre-treatment and day 30 and 60 values; however, concentrations were greater for NOV compared to E+. A reduction in serum prolactin concentration is commonly observed in cows grazing E+ fescue pastures [10,11,12,13,18,19]. There was a difference in prolactin concentrations among cows of different *DRD2* genotypes but the response to fescue was similar for A|A and G|G genotypes. Campbell et al. [29] identified the *DRD2* SNP suggested that A|A genotype may provide resistance to fescue toxicosis. They hypothesized that spring calving herds should have a higher prevalence of A allele. Our results showed that the A and G allele were about equally distributed in our Angus spring-calving cow herd (0.52 A allele and 0.48 G allele). The majority of our cows were heterozygous (56%) for the *DRD2* SNP with smaller percentages in the homozygous condition (24% A|A, 20% G|G). In this study, there was no interaction between maternal genotype and fescue endophyte treatment for serum prolactin concentrations indicating a lack of genetic association with prolactin values in cows grazing stockpiled E+ fescue. Others have also examined other single SNPs and found some associations with XK, Kell blood group complex subunit-related family member-4 (XKR4) and serum prolactin levels [35], prolactin gene and calving interval [36], and cytochrome P450 and lower milk production [37]. Selection for a trait like fescue resistance likely involves multiple genes and therefore the use of a single SNP marker may not be as successful [38]. A commercial test, T-Snip™ (AgBotanica, Columbia, MO, USA), is now available that provides a tolerance index for fescue toxicosis. Galliou et al. [39] found a relationship between T-Snip score and cow performance when grazing E+ tall fescue. The benefits of genetic markers for tolerance to fescue toxicosis would be advantageous for tall fescue based grazing systems, but more research is needed in this area.

Serum NEFA and cholesterol concentrations were measured at day 30 and 90 of the grazing endophyte type study to examine changes in metabolism that may be associated with fescue endophyte type or maternal genotype. Non-esterified fatty acids are released into the bloodstream upon lipolysis of adipose triglycerides and elevated levels are associated with negative energy balance in late gestation to early lactation in dairy cattle [40]. Serum NEFA values after 30 days of grazing were higher in A|A cows grazing E+ pastures but unchanged in G|G. At 90 days of treatment, NEFA concentrations were elevated in cows grazing E+ regardless of genotype. Niederecker et al. [41] also reported elevated NEFA values in cows grazing stockpiled tall fescue versus summer baled fescue hay after 56 to 99 days of feeding during late gestation. McArt et al. [40] found that cows with elevated NEFA had longer time to pregnancy, lower milk yield and increased culling rates. Cholesterol concentrations were higher for NOV than E+ at day 30 and 90 of fescue treatment. Others have shown that serum cholesterol content is lower in steers grazing E+ tall fescue pasture versus endophyte-free [42] or novel endophytes [43]. Stuedemann et al. [44] also reported lower plasma cholesterol concentrations in cows grazing E+ fescue and that cholesterol values were negatively associated with rate of N fertilization. The cows grazing high N fertilized E+ pastures had the lowest cholesterol levels and highest incidence of fat necrosis [44]. Cholesterol is the precursor for steroid hormones and higher cholesterol concentrations are associated with shorter intervals from calving to conception and successful pregnancy [45].

It is well documented that grazing of E+ forage results in production losses [1,2,3,5,25]. Steers grazing ergot alkaloid-producing endophyte-infected tall fescue gained 30% to 70% less on compared to cattle consuming an endophyte-free or novel endophyte tall fescue [3,20,21,46]. In this study, cows grazing E+ fescue had lighter body weights and 30% lower average daily gains during the last trimester of gestation compared to NOV. Others [12,13,18,19] have shown similar results with lower body weight gains in cows grazing E+ fescue compared to endophyte-free or novel endophyte. Reduction in BW gain for cattle consuming toxic fescue has been attributed to a number of things, but most often associated with reduced intake [9,47]. Peters et al. [12] found that forage intake was similar in the initial 28 d even though body weight loss was not. In this study, calf birth weight did not differ between fescue endophyte types even though cow body weight gain was reduced. Similarly, others have also reported no change in calf birth weight when cows grazed E+ fescue prior to calving [19,48,49]. In contrast, others have shown that calves or lambs born to dams exposed to endophyte-infected tall fescue during gestation have reduced birthweights by 5% [18], 15% [50,51] or 36% [52]. Britt et al. [50] found that exposure to ergot alkaloids during late gestation had the greatest impact on fetal development with little to no effect when fed during mid-gestation in sheep. Exposure to ergot alkaloids during late gestation reduced fetal lamb muscle and organ weights resulting in asymmetrical growth, which is indicative of intrauterine growth restriction (IUGR) [53]. These reductions in fetal growth appear related to vasoconstrictive events caused by ingestion of the ergot alkaloids, specifically ergovaline and ergovalinine [54]. In cattle, the placenta continues to increase in weight until near term [55,56] which is unlike the sheep where placental mass is established by 90 d of gestation [57]. Greenwood et al. [58] suggests that these differences in placental growth may explain why cattle appear less sensitive to under nutrition than sheep. Placental weight and birth weight are highly correlated in beef cattle [59,60]. However, in this study, cow weight gain and body weight were reduced when grazing E+ fescue pastures without any change in calf birth weight. Body condition score was lower for A|A cows grazing E+ than NOV; however, in G|G cows BCS was similar on NOV and E+ but BCS was lower than A|A cows grazing NOV. Hair coat score increased over time in both NOV and E+ but cows grazing E+ fescue had higher HCS on d 60 and 90 than NOV. Gray et al. [61] reported that cows who shed their winter coat before June 1 wean heavier calves. Real-time ultrasound measures of rump fat thickness and gluteus muscle depth at the beginning and end of the study showed that subcutaneous fat thickness was unchanged, but the change muscle depth was greater for E+ than NOV. Ultrasound rump fat and muscle depth measures are useful to monitor changes in body composition in cows grazing toxic tall fescue [62]. These results suggest the cows grazing E+ fescue during the last trimester of rapid fetal growth had reduced body weight gains that lead to mobilization of muscle tissue in order to provide nutrients for fetal growth.

This grazing study was continued through the first 30 days of lactation, which is a period of highest milk production [23,24] when the calf is almost solely dependent on the dam. Grazing E+ fescue during the last trimester and first 30 days of lactation lowered milk production by 15% compared to NOV. Milk production was also greater for G|G genotypes than A|A; however, this was consistent across endophyte treatments. Brown et al. [63] reported a 43% reduction in milk production for Angus cows grazing E+ fescue pastures compared to bermudagrass from d 61 to 200 postpartum. Peters et al. [12] found a 22% reduction in milk production of cows grazing E+ versus endophyte-free tall fescue pastures from birth to weaning. In contrast, Burke et al. [49] did not observe differences in milk production but instead found higher milk fat in cows grazing E+ fescue compared to endophyte-free. Milk production, measured at 70 days of age, did not differ in cows that grazed E+ or NOV in late gestation only [49]. Calf gains during this first 30 days period were lower for E+ than NOV. Feeding toxic endophyte-infected seed to ewes during mid and late gestation reduced milk production by 59 to 82% and reduced pre-weaning growth rates [51], which altered subsequent puberty development and post-natal growth [64]. In addition, cow calving rate was lower in cows that grazed E+ fescue during late gestation and early lactation. Calving interval from year 1 to 2 in this study did not differ among fescue treatments or genotypes. Caldwell et al. [19] also observed reduced calving rates when spring calving cows grazed E+ tall fescue compared to novel endophytes. Calf weaning weights or adjusted weaning weight for cow age and sex did not differ by endophyte type or maternal genotype. However, the adjusted 205-day weight was lower for calves whose dams grazed E+ tall fescue during late gestation and early lactation compared to NOV. Others also found that 205-day weights were lower for calves whose dams grazed E+ fescue from birth to weaning [12] and in spring-calving cows [19].

## 4. Conclusions

Grazing E+ fescue pastures during the last trimester of gestation and first 30 day of lactation reduced cow body weight gain but did not alter calf birth weight compared to NOV. Milk production and calf ADG during the first 30 day of lactation were lower for E+ than NOV. Calving rate was reduced but no calving interval for E+ cows. Adjusted 205-day weight of calves was lower in those grazing E+ with their dams compared to NOV. There were no interactions between *DRD2* genotype and fescue endophyte type indicating that genotype was not associated with response to E+ fescue in this study. Overall, grazing E+ tall fescue pastures during late gestation and early lactation appeared to alter the priority of nutrient utilization of the cows to adapt to reductions in gain during the time period of rapid fetal growth. Early milk production was lower for E+, which reduced early calf gains that remained to 205-day of age.

## 5. Materials and Methods

All animal experimental procedures were reviewed and approved by the Clemson University Institutional Animal Care and Use Committee (AUP 2017-025) on 22 May 2017. All animal experiments were conducted at the Clemson University Piedmont Research and Education Center (PREC), Pendleton, SC.

### 5.1. Genotyping

Hair follicles (5–10) were collected from tail switches of all cows in the Angus cow herd at Piedmont REC (n = 227; 3–7 year of age) and DNA was extracted using Quick Extract™ DNA extraction solution (Epicentre, Madison, WI, USA). The Extracted DNA was shipped to University of Tennessee for *DRD2* SNP genotyping (rs41749780) according to Campbell et al. [29]. Each cow was one of three possible genotypes: A|A, A|G or G|G. Dams with homozygous alleles (A|A and G|G) were used in this study over a two-year period.

### 5.2. Design

Cows were pregnancy checked by ultrasound via rectal palpation at day 95 after timed AI by an experienced technician. Pregnant cows (n = 75, 3 to 7 year of age, 608 ± 4.9 kg BW) were randomly assigned to fescue endophyte type: (1) endophyte-infected ergot alkaloid producing tall fescue (E+; Kentucky 31) or (2) novel endophyte-infected nontoxic tall fescue (NOV, Texoma MaxQ II, Pennington Seed, Madison, GA, USA) within maternal (A|A, n = 38 and G|G, n = 37) genotype during the last trimester of pregnancy (90 days) and first 30 days of lactation. Cows remained in the same fescue endophyte type treatment during year 2. Each year, there were two pasture replicates (10 ha pastures/rep; 2 rep/fescue type) per fescue endophyte type. Cows were maintained on non-fescue pastures during the first and second trimester of gestation when not on fescue endophyte treatments. For this study, cows grazed stockpiled fescue pastures with either endophyte-infected, toxic (E+) or endophyte-infected, novel (NOV) tall fescue. Pastures were fertilized 15 days prior to cows being allocated to treatment with 67.7 kg of nitrogen per ha. A strip grazing management practice was implemented through the duration of the project. Paddocks were monitored visually twice weekly and cattle were allotted additional forage when herbage availability dropped below 2000 kg/ha. When all available forage in stockpiled pastures was below 2000 kg/ha, hay of each endophyte treatment type was fed free choice in each paddock. Prior to the start of the grazing experiment each year, tiller samples were taken and sent to Agrinostics (Watkinsville, GA, USA) to determine endophyte infection levels. All pastures used in this study had > 90% endophyte infection rates.

### 5.3. Forage Analysis

Grab samples of forage were collected during the time period when cattle were grazing the endophyte treatments (late gestation to early lactation) from each paddock when cattle were moved to a new strip of grazing and hay samples collected before feeding. All forage and hay samples were freeze dried and ground using a 1-mm screen Wiley cutting mill (Arthur H. Thomas, Philadelphia, PA, USA). Forage samples were composited on a monthly basis. In year 1, forages and hay were analyzed for total ergot alkaloid infection concentrations (Agrinostics Limited Co., Watkinsville, GA, USA). As ergovaline and its epimer ergovalinine are the most informative ergots involved in vasoconstriction [5,8], we developed an assay to measure concentration of these ergopeptines to obtain more specific information. This assay was not available in year 1 but was developed and utilized in year 2 of this experiment. In year 2, forage and hay samples were analyzed for ergovaline and ergovalinine concentrations by LC-MS/MS at the Multi-User Analytical Laboratory (MUAL) at Clemson University.

For quantification of ergovaline and ergovalinine concentrations, freeze dried forage and hay samples were extracted according to Guo et al. [65]. A tall fescue seed extract was provided by J. L. Klotz and used to establish a calibration range from 0.78 to 100 ng/mL to quantify ergovaline and ergovalinine [66]. Ergotamine tartrate (Sigma Chemical Co., St. Louis, MO, USA) was used as an internal standard. Samples were analyzed using a Thermo Orbitrap Fusion™ Tribrid™ Mass Spectrometer (Thermo Fisher, Waltham, MA, USA) with MS1 (ESI positive, 60,000 resolution, scan range 220–1200 m/z) and MS2 (HCD & CID on targeted mass of 25 ergot alkaloids in orbitrap at 15,000 resolution) in the Clemson University Multi-User Analytical Laboratory and Metabolomics Core. The column was Phenomenex Kinetex XB-C18 with 1.7 μm, 100A, 150 × 2.1 mm (Phenomenex, Torrance, CA, USA). Solvent A was 5 mM ammonium bicarbonate and solvent B was 90% acetonitrile with 5 mM ammonium bicarbonate. Quality control checks were performed using instrument blanks, method blanks to determine recovery, and instrument quality control to assess relative standard deviation of 12.5 ppb ergovaline standard. The percent recovery for ergovaline, ergovalinine, and ergotamine were 98, 95, and 100%, respectively. Compound detection was performed using the extracted ion chromatogram for targeted analytes within 2 ppm mass error in Skyline software. Quantification of ergovaline and ergovalinine was performed using linear external calibration curves (0.78 to 100 ng/mL. The method limit of detection for ergovaline/ergovalinine in grass and hay was 11.414 ng per g freeze-dried plant tissue (instrument LOD of 0.78 ppb).

### 5.4. Blood Samples

Blood was collected on cows at day 0, 30, 60, and 90 of fescue endophyte treatment in 10 mL vacutainer serum tubes (Covidien Ltd., Monoject; Dublin, Ireland) via the coccygeal vein. All serum samples were allowed to clot and centrifuged at 2000× *g* for 20 min at 4 °C to obtain serum. Serum was stored at −20°C for subsequent prolactin, non-esterified fatty acid (NEFA), and cholesterol analyses. Prolactin concentrations were measured using RIA procedures of Bernard et al. [67]. The intra- and inter-assay coefficients of variation were 5.59 and 5.75 %, respectively. Non-esterified fatty acids on day 30 and 90 were analyzed using NEFA ELISA kit (BiooScientific, Perkin-Elmer, Waltham, MA, USA) with an intra- and inter-assay coefficient of variation of 6.42% and 5.56%, respectively. Serum cholesterol was extracted in duplicate using the method of Chauveau-Duriot et al. [68] and samples had an intra- and inter-assay coefficient of variation of 5.29% and 6.00%, respectively. Cholesterol content on day 30 and 90 was analyzed by gas chromotography (Agilent 6850, Santa Clara, CA, USA) using a Restek Rxi-5ms column (15 m, 0.25 mm ID, 0.25 μm, catalog no. 13420; Restek, Bellefonte, PA, USA) according to manufacturer.

### 5.5. Animals

Body condition scores (BCS 1–9 with 1 = severely emaciated to 9 = very obese) [69] and hair coat scores (HCS 1–5 with 1 = slick, shiny hair coat to 5 = > 50% of body covered with old, unshed hair) [70] were collected on day 0, 30, 60, and 90 of fescue endophyte treatment. Scores for BCS and HCS were recorded by the same two technicians during the study and average for each cow. Real-time ultrasound measures of rump fat and gluteus medius muscle depth were collected on day 0 and 90 by the same technician using an Aloka 500V and 17.2 cm linear transducer. The image was captured and interpreted using BioSoft Toolbox for beef software (Biotronics, Inc., Ames, IA, USA).

At parturition, calves were processed within 12 h of birth at 800 and 1600. Data collected included date, birth weight, and sex. Calves were ear tagged, tattooed, and males were castrated. Calves were injected intramuscularly with vitamin A (500,000 IU) and D (75,000 IU; VetOne, MWI Veterinary Supply Co., Boise, ID, USA). To estimate milk production, weigh-suckle-weigh technique was utilized at 30 days post-calving [24]. Calves were separated from their dams for 3 h and then allowed to nurse the dams dry. Then, they were separated from the dam again and held for 6 h. After the separation, calves were weighed. The calves were allowed to nurse the dam and then reweighed immediately. The difference in weights between post- and pre-suckling were assumed to represent milk production in that 6 h period and was multiplied by 4 to represent a 24-h period. In April, all cows were synchronized using the fixed-time AI (TAI) 7-day Co-Synch + CIDR protocol for timed AI to a single sire (Connealy Mentor 7374, Select Sires, registration # 15832714; Spring Hill, TN, USA). At two weeks post TAI, clean up bulls were added and cows were exposed to the clean-up bulls for 70 d. Calves were vaccinated for clostridial agents (One Shot Ultra 7; Zoetis, Parsippany, NJ, USA), bovine rhinotracheitis parainfluenza 3 vaccine (TSV-2, Zoetis), and leptospirosis (Leptoferm-5, Zoetis) in May at 4 month of age. Cows were pregnancy checked by ultrasound via rectal palpation at d 95 after TAI by an experienced technician. Cows that were not pregnant in year 1 were sold. Estimated breeding dates were calculated based on rectal palpation results. Calves were weaned on September 11, 2018 and August 30, 2019 and body weight obtained. Adjusted birth and weaning weights were calculated according to the Beef Improvement Federation Guidelines [30] based on age of the dam and sex of the calf. Adjusted 205-day weights were also calculated [30].

### 5.6. Statistics

The univariate procedure of SAS (SAS Inst. Inc., Cary, NC, USA) was used to test all variables for normality. Serum prolactin concentrations were not normally distributed and were log-transformed for data analysis. Ergot alkaloid values were not normally distributed and were square root transformed for statistical analysis. Serum prolactin and ergot alkaloid concentrations results are presented as non-transformed for easier understanding and interpretation. For forage and hay ergot alkaloid data, paddock was the experimental unit. Data were analyzed using mixed procedure with fescue forage type, month, and two-way interaction in the model. For animal data, a 2 × 2 factorial with two-factors of fescue endophyte type (E+ vs. NOV) and maternal DRD2 genotype (A|A vs. G|G) in a split plot design. A mixed model was developed with endophyte type, DRD2 genotype and the 2-way interaction in the model as fixed effects and two different error terms applied. The error term for testing endophyte type was the random effect of paddock within endophyte type by year and the error term for testing DRD2 genotype and its interactions with endophyte type was the random effect of cow within DRD2 and endophyte type combination. The analysis was performed using the GLIMMIX procedure of SAS (SAS Inst. Inc., Cary, NC, USA). For repeated measures analyses, time and all interactions with time were included in the model. Least square means were generated and separated using a Fisher’s LSD when the *F*-test was significant for year, fescue endophyte type, maternal genotype and or the interaction. Significance was determined at *p* < 0.05, and trends denoted when *p* > 0.05 to *p* < 0.10. When the interaction was non-significant (*p* > 0.05), main effects of endophyte type or DRD2 genotype are shown in the results. When the interaction was significant (*p* < 0.05), simple effects of endophyte type by DRD2 genotype are shown in the results.

## Figures and Tables

**Table 1:** Total ergot alkaloid or ergovaline + ergovalinine concentrations by fescue endophyte-infected type, toxic (E+) or novel endophyte infected (NOV) pasture or hay.

	NOV	E+	SEM
Total ergot alkaloids (Year 1)			
Pasture, ng/g	184 ^b^	1938 ^a^	93.9
Hay, ng/g	277 ^b^	611 ^a^	47.2
Ergovaline/ergovalinine, (Year 2)			
Pasture, ng/g	0 ^b^	602 ^a^	80.3
Hay, ng/g	0 ^b^	477 ^a^	43.8

^a,b^ Means in the same row differ (*p* < 0.05) by endophyte type.

**Table 2:** Main effects for year, fescue endophyte type and DRD2 genotype on adjusted calf birth weight and weaning weight of calves. All interactions were non-significant (*p* > 0.05).

	Year		Fescue Type		*DRD2* Genotype		Standard Error
	1	2	NOV	E+	AA	GG	
**Cow Parameters**							
Dystocia score ^1^	1.09	1.07	1.09	1.08	1.07	1.09	0.37
Calving interval, d	-	-	382	371	375	378	23.2
Calving rate, %	-	-	85.7 ^c^	72.5 ^d^	82.5	75.7	2.90
Milk production, kg/d	11.40	10.85	12.00 ^c^	10.25 ^d^	10.03 ^f^	12.22 ^e^	4.20
**Calf Parameters**							
**at Birth**							
Birth weight, kg	39.45	36.80	38.37	37.92	39.09 ^e^	37.21 ^f^	4.94
Adj. birth weight, kg	40.42	37.07	38.94	38.54	39.59 ^e^	37.90 ^f^	4.89
Calf sex (2 = female, 3 = male)	2.59	2.55	2.59	2.56	2.61	2.53	0.50
**at Weaning**							
Weaning age, d	220.2	202.4	210.0	212.6	212.7	209.9	20.13
Actual weaning weight, kg	269.3 ^a^	247.4 ^b^	258.9	255.0	260.0	253.9	33.8
Adj. weaning weight ^2^, kg	277.0 ^a^	247.3 ^b^	264.2	260.18	264.5	259.8	33.06
Adj. 205 d wean weight ^2^, kg	258.6	252.5	259.4 ^g^	252.7 ^h^	256.6	248.5	26.79
Adj. 205 d wean ADG, kg/d	1.064	1.048	1.075 ^g^	1.037 ^h^	1.057	1.055	0.132

^1^ Dystocia score: 1 = no difficulty, no assistance required; 2 = minor difficulty, some assistance; 3 = major difficulty, usually mechanical assistance; 4 = caesarian section or other surgery; 5 = abnormal presentation. ^2^ Adjusted weaning weight was adjusted by cow age and calf sex [30]. Adjusted 205-day weight was the adjusted weaning weight calculated on an equal calf age basis of 205-day. ^a,b^ Means in the same row with uncommon superscripts differ (*p* < 0.05) by year. ^c,d^ Means in the same row with uncommon superscripts differ (*p* < 0.05) fescue endophyte type. ^e,f^ Means in the same row with uncommon superscripts differ (*p* < 0.05) by maternal DRD2 genotype. ^g,h^ Means in the same row with uncommon superscripts differ (*p* < 0.10) by fescue endophyte type.

## Data Availability

The data presented in this study are available on request from the corresponding author.
